# Chronic *Toxoplasma gondii* infection enhances β-amyloid phagocytosis and clearance by recruited monocytes

**DOI:** 10.1186/s40478-016-0293-8

**Published:** 2016-03-16

**Authors:** Luisa Möhle, Nicole Israel, Kristin Paarmann, Markus Krohn, Sabine Pietkiewicz, Andreas Müller, Inna N. Lavrik, Jeffrey S. Buguliskis, Björn H. Schott, Dirk Schlüter, Eckart D. Gundelfinger, Dirk Montag, Ulrike Seifert, Jens Pahnke, Ildiko Rita Dunay

**Affiliations:** Institute for Medical Microbiology and Hospital Hygiene, University of Magdeburg, Leipziger Str. 44, 39120 Magdeburg, Germany; Institute for Molecular and Clinical Immunology, University of Magdeburg, Magdeburg, Germany; Department of Pathology (PAT), Translational Neurodegeneration Research and Neuropathology Lab, University of Oslo (UiO) and Oslo University Hospital (OUS), Oslo, Norway; Department of Translational Inflammation Research, Institute of Experimental Internal Medicine, University of Magdeburg, Magdeburg, Germany; Helmholtz Centre for Infection Research, Braunschweig, Germany; Genetic Engineering and Biotechnology News, New York, USA; Center for Behavioral Brain Sciences (CBBS), University of Magdeburg, Magdeburg, Germany; Department of Behavioral Neurology, Leibniz Institute for Neurobiology, Magdeburg, Germany; Department of Psychiatry and Psychotherapy, Campus Mitte, Charité Universitätsmedizin, Berlin, Germany; Department of Neurochemistry and Molecular Biology, Leibniz Institute for Neurobiology, Magdeburg, Germany; Medical Faculty, University of Magdeburg, Magdeburg, Germany; German Center for Neurodegenerative Diseases (DZNE), Magdeburg, Germany; Neurogenetics, Leibniz Institute for Neurobiology, Magdeburg, Germany; University of Lübeck (UzL), LIED, Lübeck, Germany; Leibniz Institute of Plant Biochemistry (IPB), Halle, Germany

**Keywords:** Alzheimer’s disease, Toxoplasma gondii, Chronic infection, Ly6C^hi^ monocytes, Aβ clearance

## Abstract

**Introduction:**

Alzheimer’s disease (AD) is associated with the accumulation of β-amyloid (Aβ) as senile plaques in the brain, thus leading to neurodegeneration and cognitive impairment. Plaque formation depends not merely on the amount of generated Aβ peptides, but more importantly on their effective removal. Chronic infections with neurotropic pathogens, most prominently the parasite *Toxoplasma* (*T*.) *gondii*, are frequent in the elderly, and it has been suggested that the resulting neuroinflammation may influence the course of AD. In the present study, we investigated how chronic *T. gondii* infection and resulting neuroinflammation affect plaque deposition and removal in a mouse model of AD.

**Results:**

Chronic infection with *T. gondii* was associated with reduced Aβ and plaque load in 5xFAD mice. Upon infection, myeloid-derived CCR2^hi^ Ly6C^hi^ monocytes, CCR2^+^ Ly6C^int^, and CCR2^+^ Ly6C^low^ mononuclear cells were recruited to the brain of mice. Compared to microglia, these recruited mononuclear cells showed highly increased phagocytic capacity of Aβ *ex vivo*. The F4/80^+^ Ly6C^low^ macrophages expressed high levels of Triggering Receptor Expressed on Myeloid cells 2 (TREM2), CD36, and Scavenger Receptor A1 (SCARA1), indicating phagocytic activity. Importantly, selective ablation of CCR2^+^ Ly6C^hi^ monocytes resulted in an increased amount of Aβ in infected mice. Elevated insulin-degrading enzyme (*IDE*), matrix metalloproteinase 9 (*MMP9*), as well as immunoproteasome subunits β*1i/LMP2*, *β2i/MECL-1*, and *β5i/LMP7* mRNA levels in the infected brains indicated increased proteolytic Aβ degradation. Particularly, *LMP7* was highly expressed by the recruited mononuclear cells in the brain, suggesting a novel mechanism of Aβ clearance.

**Conclusions:**

Our results indicate that chronic *Toxoplasma* infection ameliorates β-amyloidosis in a murine model of AD by activation of the immune system, specifically by recruitment of Ly6C^hi^ monocytes and by enhancement of phagocytosis and degradation of soluble Aβ. Our findings provide evidence for a modulatory role of inflammation-induced Aβ phagocytosis and degradation by newly recruited peripheral immune cells in the pathophysiology of AD.

**Electronic supplementary material:**

The online version of this article (doi:10.1186/s40478-016-0293-8) contains supplementary material, which is available to authorized users.

## Introduction

Alzheimer’s disease (AD) is the most prevalent cause of dementia in the elderly, affecting more than 15 million people worldwide [1]. Although AD was first described more than a century ago, the exact pathomechanisms of this disorder are still not fully understood. According to the widely accepted amyloid cascade hypothesis [2], the amyloid precursor protein (APP) is cleaved by β- and γ-secretases, and the resulting Aβ_40_ and Aβ_42_ peptides accumulate as fibrils and plaques, due to their highly hydrophobic nature [3–5]. However, in aging individuals and in AD patients it is not overproduction, but reduced clearance of β-amyloid (Aβ) that is thought to contribute to disease progression [6]. Specific transporters at the blood–brain barrier were discovered to transport Aβ and their insufficient function leads to intracerebral Aβ accumulation and plaque formation [7–10]. Clearance by the brain’s phagocytic system is also known to play an important role in limiting disease progression. At first, microglia cells become activated, but later fall into a state of senescence, thus failing to effectively remove intracellular and extracellular Aβ – a development that is paralleled by ongoing neuroinflammation [11–13]. Ultimately, severe neurodegeneration leads to cognitive impairments and behavioral alterations [14].

The role of innate immune cells has been discussed extensively in an attempt to untangle their beneficial and detrimental contributions in brain homeostasis and pathology [15–18]. Previously, all phagocytic cells in the brain have been termed “microglia”, until it recently became evident that resident microglia develop from yolk sac macrophages and populate the CNS during early fetal development [19], whereas distinct populations of bone marrow-derived mononuclear cells can enter the adult CNS under inflammatory and even under steady-state conditions [20–22]. Furthermore, infiltrating myeloid-derived Ly6C^hi^ CCR2^+^ monocytes also give rise to brain macrophages [17, 23–25]. Subsequent differentiation of resident and recruited innate immune cells has revealed distinct contributions of each subset under various pathological conditions [22]. Resident microglia accumulate directly around Aβ plaques, and contribute to Aβ clearance in the early phase of AD development. Later on, their role becomes a detrimental one, as they promote neurodegeneration by pro-inflammatory cytokine production [26]. Interestingly, bone marrow-derived mononuclear cells recruited to the CNS contribute to repair after spinal cord injury [27] as well as Aβ uptake and plaque elimination [28–30].

Several studies have described that AD is exacerbated by repeated or chronic systemic inflammation as well as by several infections in humans and different murine models [31–36]. It has even been speculated that specific pathogens may be causative in AD [37, 38]. The most common sporadic form of AD primarily occurs in the elderly, and older age is also associated with elevated susceptibility to infectious diseases [39], and an increased probability of having accumulated dormant infections, including toxoplasmosis [40–42].

Toxoplasmosis, caused by the obligate intracellular protozoan parasite *Toxoplasma* (*T.*) *gondii*, is a worldwide zoonosis with high medical relevance [40]. Serologic prevalence data suggest that the geographical distribution of the infection varies between 20 and 90 %, depending mainly on culinary habits and hygiene. Moreover, the infection rate strongly increases with age [43], as latent *T.* *gondii* infection is associated with lifelong parasite persistence and typically remains clinically asymptomatic in immunocompetent individuals. However, recent studies – by our laboratory and others – indicate an ongoing basal inflammation in the CNS following chronic *T.* *gondii* infection in rodents [44–46]. The subtle neuromodulatory capacity of the parasite in humans and mice has also been an area of recent investigation [47]. Prominent features of cerebral toxoplasmosis in animal models are the activation of glia and recruitment of peripheral immune cells to the CNS [18, 48, 49]. Recruited Ly6C^hi^ monocytes co-expressing C-C chemokine receptor type 2 (CCR2) [50] are critical to governing acute and chronic stages of infection [18, 23, 51–53]. Principally, we have recently described the contribution of Ly6C^int^ monocyte-derived dendritic cells and Ly6C^low^ monocyte-derived macrophages to parasite control upon chronic toxoplasmosis in the CNS [23].

The role of *T.* *gondii* in the development and course of AD is currently debated. Kusbeci *et al.* reported increased seroprevalence of anti-*T.* *gondii* IgG in a small group of AD patients, suggesting an implication of the parasite in AD pathogenesis [54], but these findings could not be replicated in another study [55]. Animal studies have suggested that *T.* *gondii* infection may actually be able to prevent neurodegeneration [56, 57] and can even reduce plaque burden and prevent memory decline [58]. The latter studies suggest that *T.* *gondii* may actually play a protective rather than a detrimental role in AD. However, they do not address the underlying mechanisms of plaque reduction. In the present study, we investigated how AD progression is influenced by the boosted presence of myeloid cells in the CNS in latent toxoplasmosis.

Our data reveal that upon chronic *Toxoplasma* infection, recruited mononuclear cells are capable of ameliorating plaque burden in a murine model of cerebral β-amyloidosis through Aβ phagocytosis and increased degradation.

## Materials and methods

### Animal models

All animal experiments were approved according to German and European legislation by the local authorities. Experiments were conducted with 8 weeks old C57BL/6J mice and female and male 5xFAD mice (5xFAD/Tg6799 strain (B6SJL-Tg(APPSwFlLon, PSEN1*M146L*L286V) 6799Vas/Mmjax backcrossed for >10 generations to C57BL/6J, JAX stock#006554) [59, 60]. In this model, Aβ plaques in the brain are detectable from the age of 50 days and their number increases by age [61]. At least five animals per group in up to two independent experiments were used.

### *T. gondii* infection

*T. gondii* cysts of the ME49 type II strain were used for this study. Parasites were harvested from the brains of female NMRI mice infected intraperitoneally (i. p.) with *T. gondii* cysts 4 to 5 months earlier. Brains obtained from infected mice were mechanically homogenized in 1 mL sterile phosphate-buffered saline (PBS) and the cysts were counted using a light microscope. Two cysts were administered i. p. into 8-week-old mice in a total volume of 200 μL. This time point was chosen because at the age of 8 weeks, animals are fertile and are considered adults in this respect.

### Monocyte depletion

To specifically ablate CCR2^+^Ly6C^hi^ monocytes, 66 μg of anti-CCR2 antibody (clone MC-21, provided by M. Mack, University of Regensburg) were administered i. p. on d15, d18, d21, d24 and d27 post infection. Control mice received PBS. On d22 (12-15 h post antibody injection), blood was collected retro-orbitally to confirm depletion. Mice were sacrificed and samples were collected on d28.

### Blood and brain sampling

At the chronic stage of infection (8 weeks post infection and 4 weeks post infection for depletion and phagocytosis assays, see below), mice were deeply anesthetized and if necessary blood was drawn from the inferior vena cava using a 26G needle and syringe. Subsequently, mice were perfused intracardially with 60 ml sterile ice-cold PBS. Brains were removed and prepared accordingly for further analysis.

### Histopathology

Brain hemispheres were removed and immersed in 4 % paraformaldehyde (PFA) for several days. Paraffin-embedded, 4 μm thick sections were deparaffinized and conventionally stained with hematoxylin-eosin (H&E) stain. Immunohistochemical analysis was performed according to our previous publications [12, 13, 62–66] using a BOND-MAX (Leica Microsystems GmbH/Menarini, Germany) with antibodies against Aβ (clone 4G8, Chemicon, Germany), ionized calcium-binding adapter molecule 1 (IBA1, Wako 019–19741, Germany) to label microglia, glial fibrillary acid protein (GFAP, DAKO Z033401, Germany) to label astrocytes, NeuN (Millipore MAB377, Germany) to label neurons, and anti-Toxo (Dianova DLN-16734, Germany) to label *T. gondii*. Slides were developed using the Bond^TM^ Polymer Refine Detection kit (Menarini/Leica, Germany). For the evaluation whole tissue sections were digitized at 230 nm resolution using a MiraxMidi Slide Scanner (ZeissMicroImaging GmbH, Germany) [67].

### Immunofluorescence analysis

For immunofluorescent staining, coronal brain sections (16 μm) were prepared with a cryomicrotome (Leica, Germany). Immunolabeling with antibodies against Aβ (4G8, Chemicon, Germany), Iba1 (polyclonal, Wako) and Ly6C (ER-MP20, Acris Antibodies, Germany) were performed overnight at 4 °C after 2 min pretreatment with 98 % formic acid. Secondary antibodies goat anti-rat (Alexa Fluor 488, 1:200, Invitrogen, Germany), goat anti-rabbit (Alexa Fluor 488, Invitrogen, Germany) and goat anti-mouse (Alexa Fluor 594, Invitrogen, Germany) were used. Free floating sections were mounted with ProLong Gold with DAPI (life technologies, Germany). A Zeiss (Carl Zeiss, Germany) microscope equipped with an AxioCam HRc 3 digital camera and AxioVision 4 Software were used to analyze staining and obtain images.

Quantification of Iba1 and Ly6C association with plaques was performed using the ImageJ plot profile function (http://imagej.nih.gov/ij/). For this purpose, two perpendicular fluorescence profiles spanning 400 μm and centered over the plaques were measured in immunofluorescence stainings. At least 30 plaques from at least four different tissue sections were analyzed.

### Two-Photon image acquisition and analysis

For in vivo staining of amyloid plaques, mice were i. p. injected with 10 mg kg^−1^ methoxy-X04 (Tocris Bioscience) in 5 % DMSO/95 % NaCl (0.9 %) 12 h before brain harvesting and two-photon image acquisition.

Brains were placed under microscopy coverslips for *ex vivo* microscopy using a Zeiss LSM 710 (Carl Zeiss, Jena, Germany) equipped with a MaiTai DeepSee 2-Photon laser (Spectra-Physics, Darmstadt, Germany) tuned at 800 nm. Fluorescence emission was split using dichroic mirrors and detected using non-descanned detectors. Methoxy-X04 fluorescence was read out at 450-490 nm. Fluorescence signal acquired above 520 nm was considered autofluorescence. Confocal stacks spanning at least 50 μm were collected with a z-spacing of 4 μm using a W Plan-Apochromat 20x water immersion objective with a numerical aperture of 1.0.

Images were processed and superimposed using the Imaris (Version 7.7., Bitplane, Zürich, Switzerland) software. Methoxy-X04-positive objects co-localizing with blood vessels (identified by different tissue autofluorescence) were manually excluded from the analysis. Plaques were automatically detected and quantified in three dimensions using the measurement package of the Imaris software.

### Enzyme-linked immunosorbent assay (ELISA) and preparation of protein extracts

Freshly harvested brain tissue was snap-frozen in liquid nitrogen and stored at −80 °C. Aβ_42_ was quantified in whole brain hemispheres by ELISA against human Aβ_42_ (Thermo Fisher Scientific, Germany). For total Aβ_42_ quantification, brains were homogenized in 5 M guanidine hydrochloride buffer (GuHCl) (5 M GuHCl in 50 mM Tris/HCl, pH 8.0) and incubated for 4 h at room temperature on a shaker. For discrimination between small (soluble) and large (insoluble) Aβ_42_ aggregates, brains were homogenized in carbonate buffer (100 mM Na_2_CO_3_ in 50 mM NaCl, pH 11.5, 20 μl/mg) and centrifuged. The supernatant was mixed with 8 M GuHCl (in 82 mM Tris/HCl, 0.6 ml GuHCl/1 ml carbonate buffer) to obtain the soluble Aβ_42_ fraction. The pellet was dissolved in 5 M GuHCl (10 μl/mg) and incubated 3 h at RT on a shaker to obtain the insoluble Aβ_42_ fraction. Prior to ELISA analysis, each extract (total, soluble and insoluble) was diluted with BSAT-PBS (5 % bovine serum albumin in PBS with 0.03 % Tween-20 and protease inhibitor (Roche, Germany)) according to the expected Aβ_42_ content. ELISA was then performed according to the manufacturer’s instructions.

### RT-PCR from whole-brain homogenates

After removal, tissue samples from brains were immediately transferred to RNA later (QIAgen, Germany). Total RNA was isolated as previously described with the peqGOLD HP Total RNA Kit (peqlab, Germany) including an on-membrane DNase I digestion (peqlab, Germany) [18].

Relative gene expression was determined similar to previous descriptions [18, 68] using TaqMan® RNA-to-C_T_^TM^ 1-Step Kit (life technologies, Germany). Reactions were developed in a LightCycler® 480 Instrument II (Roche, Germany). Reverse transcription was performed for 15 min at 48 °C followed by 10 min at 95 °C. Subsequently, 45 amplification cycles were run, comprising of denaturation at 95 °C for 15 s and annealing/elongation at 60 °C for 1 min. TaqMan® Gene Expression Assays (life technologies, Germany) were used for mRNA amplification of HPRT (Mm01545399_m1), IDE (Mm00473077_m1), (IL10, Mm00439616_m1), MMP9 (Mm00442991_m1), NEP (MME, Mm00485028_m1), PSMB8/LMP7 (Mm00440207_m1), PSMB9/LMP2 (Mm00479004_m1) and PSMB10/MECL-1 (Mm00479052_g1). HPRT mRNA expression was chosen as reference for normalization and target/reference ratios were calculated with the LightCycler® 480 Software release 1.5.0 (Roche, Germany). Resulting data were further normalized to values of appropriate control groups.

### RT-PCR from sorted cell populations

Isolated brain single cell suspensions were surface stained as described below and sorted on a BD FACSAria^TM^ III. After sorting, cells were pelleted, any remaining liquid was removed and cells were frozen at −80 °C. Total RNA was isolated using the RNeasy® Mini Kit (QIAgen, Germany). cDNA was synthetized with the iScript™ cDNA Synthesis Kit (BIO-RAD, Germany). Relative gene expression was measured using the TaqMan® Universal PCR Master Mix (Applied Biosystems, Germany). TaqMan® Gene Expression Assays (life technologies, Germany) and data analysis was the same as for RT-PCR from whole-brain homogenates.

### Cell isolation

For mononuclear cell isolation brains were homogenized in a buffer containing 1 M Hepes pH 7.3 and 45 % glucose and then sieved through a 70 μm strainer as published previously [18]. The cell suspension was washed and re-suspended in 10 mL 75 % Percoll (GE Healthcare, Germany) in PBS and over layered with 10 mL 25 % Percoll in PBS and 5 mL PBS. The gradient was centrifuged for 45 min at 800 g without brake. Cells were recovered from the 25 %/75 % interphase, washed and used immediately for further experiments.

### Surface staining

Single cell suspensions were incubated with an anti-FcγIII/II receptor antibody (clone 93) to block unspecific binding and Zombie Violet^TM^ (Biolegend, Germany), a fixable viability dye. Thereafter, cells were stained with fluorochrome conjugated antibodies against cell surface markers: CD45 (30-F11, eBioscience, Germany), CD11b (M1/70, eBioscience, Germany), Ly6G (1A8, BD Biosciences, Germany), Ly6C (HK1.4, eBioscience, Germany), CCR2 (475301, R&D, USA), TREM2 (237920, R&D, USA), CD36 (HM36, Biolegend, Germany), in FACS buffer (PBS containing 2 % FCS and 0.1 % NaN_3_) for 30 min on ice and then washed and fixed in 4 % paraformaldehyde (PFA) for 10 min.

### *Ex vivo* phagocytosis assay

Animals were sacrificed 4 weeks post infection and single cell suspensions were prepared as described above. Live cells remained unstained by the Zombie Violet^TM^ (Biolegend, Germany) viability dye and were sorted on a BD FACSAria^TM^ III. 150,000 cells were seeded into each well of a 96-well round bottom plate and allowed to settle down for 1 h in an incubator (37 °C, with 5 % CO_2_ and 70 % humidity) before HiLyte Fluor^TM^ 488-labeled, HFIP monomerized Aβ_42_ (Eurogentec, Belgium) was added to a final concentration of 500nM. Cells were incubated for 6 h, washed, surface stained and measured by conventional and imaging flow cytometry.

### Conventional flow cytometry

Cell acquisition was performed on a BD FACS Canto^TM^ II flow cytometer. Data were analyzed using FlowJo software (TreeStar).

### Imaging flow cytometry

Data were acquired with FlowSight^TM^ (EMD Millipore, USA) with a 20x objective and analyzed using IDEAS software version 6.0. At least 52,000 (control animals) or 500,000 (*T. gondii* infected animals) cells were acquired for each sample and gated to select images with single cells in good focus (by bright field area/aspect ratio and gradient root mean square of the bright-field image, respectively). To analyze internalization, the erode mask was used on the bright field picture of Aβ^+^ cells to remove 2 pixels from the edges of the starting mask and to define the inner part of the cell. Then, the internalization feature was used to define the ratio of intensity of the Aβ signal between the inside of the cells (defined by the erode mask) and the intensity of the total cell. While cells with little internalization have negative scores, cells with high internalization have positive scores. Here, an internalization > 0 was defined as intermediate to high internalization. Compensation matrix generated by single-color compensation controls was used to correct spectral overlap. Representative pictures from cell populations were chosen.

### Statistical analysis

Results are presented as mean + standard error of the mean (SEM). Statistical analysis was performed with Prism version 6 (GraphPad Software, USA). Different tests were used to compare values, namely Mann–Whitney U test (plaque numbers and volume), Fisher’s LSD test (results with multiple comparisons) and Student’s t test (results with one comparison). *p* values of *p* ≤ 0.05 were considered statistically significant.

## Results

### *T.* *gondii* infection reduces the plaque burden in 5xFAD mice

**Fig. 1:**
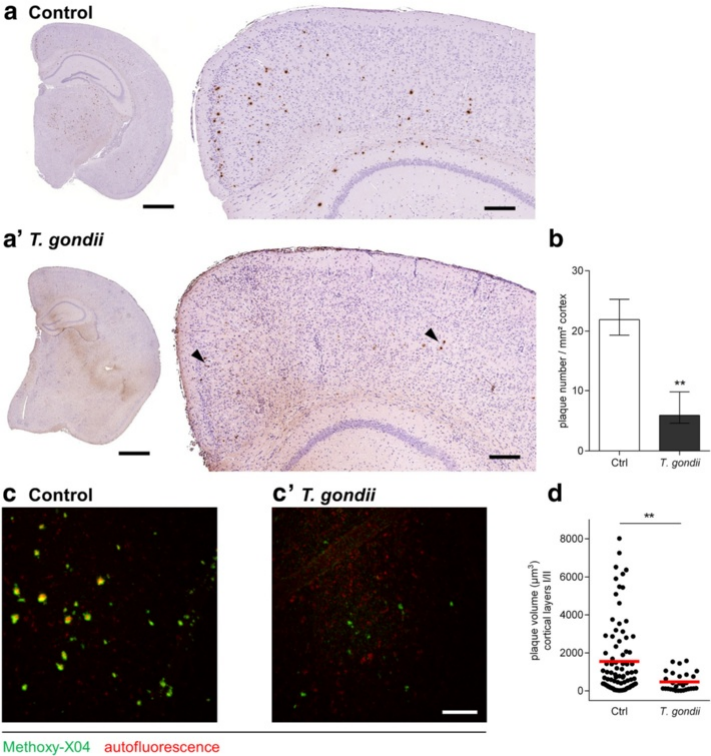
*T.* *gondii* infection leads to reduced plaque burden in 5xFAD mice. **a**, **a’** show representative cortex regions with immunohistochemical labeling against Aβ from (**a**) non-infected and (**a’**) *T.* *gondii* infected 5xFAD mice. Arrow heads point towards remaining plaques in infected animals. Scale bars represent 1000 μm (*left*) and 200 μm (*right*). **b** Quantification of cortical Aβ plaques. Data is displayed as mean ± SEM. Significance as determined by unpaired t test with Welsh‘s correction is indicated. **p ≤ 0.01. **c**, **c’** *Ex vivo* two-photon micrographs of the cerebral cortex layers I and II of (**c**) control and (**c’**) *T.* *gondii* infected 5xFAD mice injected with Methoxy-X04. Amyloid plaques stained with Methoxy-X04 are represented in green, autofluorescence is shown in red. One representative z-stack projection spanning at least 50 μm depth is shown for each condition. Scale bar, 100 μm. **d** Plaque volume in cortical layers I and II determined from at least five *ex vivo* two-photon micrographs shown in (**c**, **c’**). Each symbol represents one plaque and bars represent the mean, statistical significance is denoted by asterisks. **p ≤ 0.01

Infection with *T.* *gondii* led to reduced plaque burden as determined by immunohistological staining of Aβ plaques in 5xFAD mice (Fig. 1a and a’). Quantification revealed significantly lower plaque numbers in the cortex of mice infected with *T.* *gondii* as compared to transgenic control mice (control 21.9 plaques/mm^2^, *T.* *gondii* 5.9 plaques/mm^2^, *p* < 0.006, Fig. 1b). This observation was further confirmed by *in situ* two-photon microscopy, which was technically restricted to the observation of cortical layers I and II (Fig. 1c and c’). Here, methoxy-X04 stained plaque volumes of the remaining amyloid plaques were significantly reduced in *T.* *gondii* infected animals (controls 1548 ± 205 μm^3^, *T.* *gondii* 466 ± 92 μm^3^, *p* < 0.002, Fig. 1d).

**Fig. 2:**
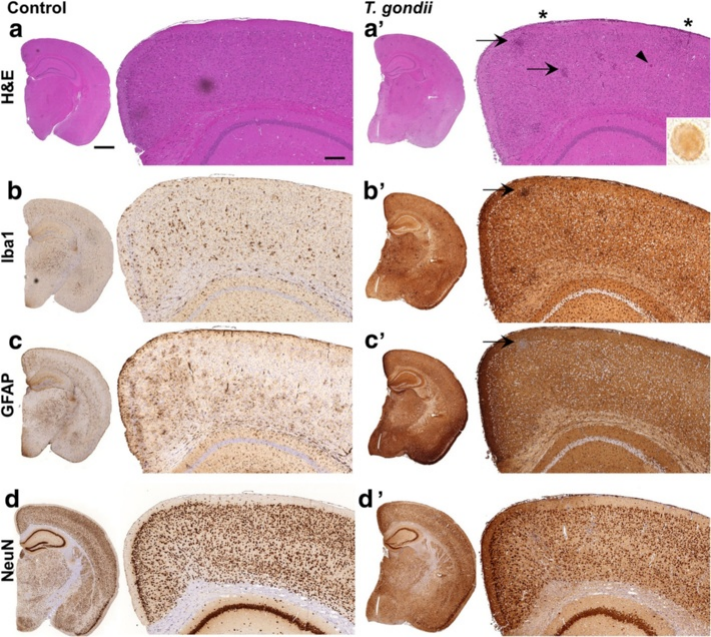
*T.* *gondii* infection induces histopathological changes and activation of microglia. **a**, **a’** Hematoxylin and eosin (H&E) stained coronal sections from (**a**) non-infected and (**a’**) *T.* *gondii* infected 5xFAD mice. * denotes meningeal infiltrates, arrows show inflammatory foci, the arrow head highlights a *T.* *gondii* cyst. The inset shows a cyst stained with an antibody against *T.* *gondii*. **b**–**d’** Representative pictures of immunohistochemical stainings against (**b**, **b’**) Iba1, (**c**, **c’**) GFAP, and (**d**, **d’**) NeuN of coronal brain sections from (**b**, **c**, **d**) non-infected and (**b’**, **c’**, **d’**) *T.* *gondii* infected 5xFAD mice. Scale bars represent 1000 μm (*left column*) and 200 μm (*right column*)

Using morphological methods, we detected substantial changes in the cortex and subcortical regions of infected mice such as inflammatory lesions (Fig. 2a’), extensive ionized calcium-binding adapter molecule 1 (Iba1)-reactivity (Fig. 2b’), due to the activation of microglia and myeloid cell infiltration, as well as glial fibrillary acid protein (GFAP)-positive astrogliosis (Fig. 2c’). Non-infected 5xFAD mice presented with known brain histology (Fig. 2a) and only minor activation of resident glia cells (Iba1 and GFAP staining, Fig. 2b, c) [59]. Specific immunodetection for neurons (NeuN labeling) revealed no alterations in the number of neurons in the cortex of infected versus control mice (Fig. 2d, d’). The slightly darker shading in the infected brain is due to increased background staining as previously seen in this infection model in wildtype C57BL/6J mice [18]. Occasionally, *T.* *gondii* cysts were detectable (Fig. 2a, inset).

Noting the vast activation of microglia and astrocytes in the infected animals, we have to potentially take into account additional specific and unspecific immunological responses as further factors for the reduction of Aβ [69, 70].

### Recruited monocytes express high CCR2, intermediate TREM2 and CD36

**Fig. 3:**
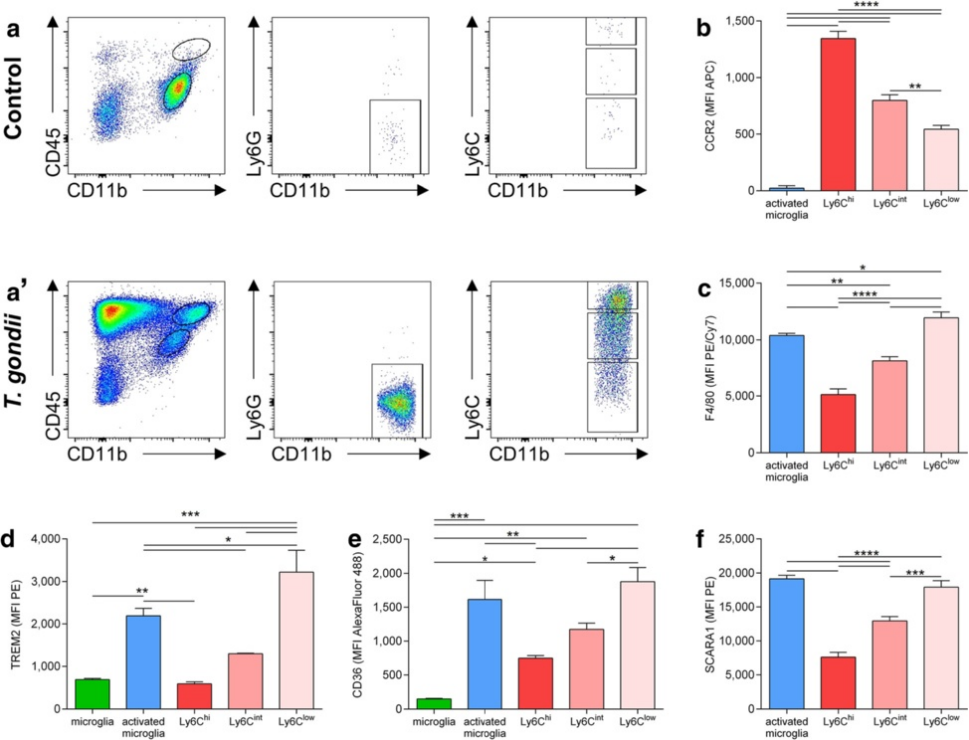
Myeloid-derived mononuclear cells are recruited to the brain upon *T.* *gondii* infection and express phagocytosis related surface molecules. Mononuclear cells were isolated from 5xFAD mouse brains and subjected to flowcytometric analysis. **a**, **a’** Representative pseudocolor plots are shown for (**a**) non-infected and (**a’**) infected 5xFAD animals and demonstrate the recruitment of CD45^hi^CD11b^hi^Ly6G^neg^Ly6C^+^ cells to the brain upon *T.* *gondii* infection. After gating cells by their forward and side scatter properties, excluding doublets and dead cells (not shown), we used CD45 and CD11b expression to discriminate between resting microglia (**a**, bottom elliptic gate) or activated microglia (**a’**, bottom elliptic gate), respectively, and myeloid cells (**a’**, top elliptic gate). From myeloid cells, Ly6G^+^ neutrophils were excluded and the Ly6C expression of the remaining CD11b^hi^Ly6G^−^ cells was used to gate Ly6C^hi^, Ly6C^int^ and Ly6C^low^ mononuclear cells. **b**–**f** We compared the surface expression of CCR2, F4/80, TREM2, CD36, and SCARA1 between resting microglia, activated microglia and myeloid-derived mononuclear cell subsets. The median fluorescence intensity (MFI) for each marker and population is displayed as mean + SEM. Significance levels (*p* values) determined by Fisher’s LSD test are indicated. *p ≤ 0.05, ***p ≤ 0.001, ****p ≤ 0.0001

Activation of resident microglia is complemented by recruitment of immune cells from the periphery to the CNS. Collectively, resident and recruited cells form a robust immune response to control *T.* *gondii* [18]. Among recruited immune cells, the myeloid compartment plays an important role in cerebral toxoplasmosis [71–73]. In the following, we focused our analysis on three subsets of myeloid-derived CD45^hi^ CD11b^hi^ Ly6G^neg^ CCR2^+^ mononuclear cells, the CCR2^hi^ Ly6C^hi^ monocytes, Ly6C^int^ cells and Ly6C^low^ macrophages (gating strategy depicted in Fig. 3a, a’). These subsets of myeloid cells were chosen because age-related defects in the Ly6C^hi^ monocyte population have been linked to cognitive decline in a murine AD model [74]. First, we detected increased numbers of engrafted Ly6C^hi^, Ly6C^int^ and Ly6C^low^ cells in the brains of *T.* *gondii* infected 5xFAD mice compared to non-infected mice (Fig. 3a, a’).

Subsequently, we determined the expression of certain phagocytosis-related surface molecules and quantified the MFI by flow cytometry: Triggering Receptor Expressed on Myeloid cells 2 (TREM2), CD36, and Scavenger Receptor A1 (SCARA1). We compared their expression on resident and myeloid-derived mononuclear cells and the pattern was similar for all three markers: We found the highest expression of TREM2, CD36 and SCARA1 on Ly6C^low^ F4/80^hi^ macrophages and activated microglia and a lower expression on Ly6C^hi^ CCR2^hi^ monocytes and resting microglia. Ly6C^int^ mononuclear cells expressed intermediate amounts of TREM2, CD36 and SCARA1 on their surface (TREM2: microglia 688 ± 25, activated microglia 2191 ± 179, Ly6C^hi^ 595 ± 48, Ly6C^int^ 1298 ± 13, Ly6C^low^ 3222 ± 511, Fig. 3d; CD36: microglia 152 ± 8, activated microglia 1616 ± 281, Ly6C^hi^ 749 ± 37, Ly6C^int^ 1175 ± 92, Ly6C^low^ 1879 ± 208, Fig. 3e; SCARA1: activated microglia 15977 ± 3220, Ly6C^hi^ 6319 ± 1395, Ly6C^int^ 10805 ± 2221, Ly6C^low^ 14964 ± 3088, Fig. 3f).

Resident microglia activation was confirmed by increased CD11c, MHC I and MHC II levels as measured by flow cytometry (data not shown). Thus, our data indicate that cerebral toxoplasmosis induces the recruitment of different myeloid-derived mononuclear cell subsets to the CNS that may contribute to the removal of Aβ by activated microglia.

### Myeloid-derived mononuclear cells p**h**agocytose Aβ

**Fig. 4:**
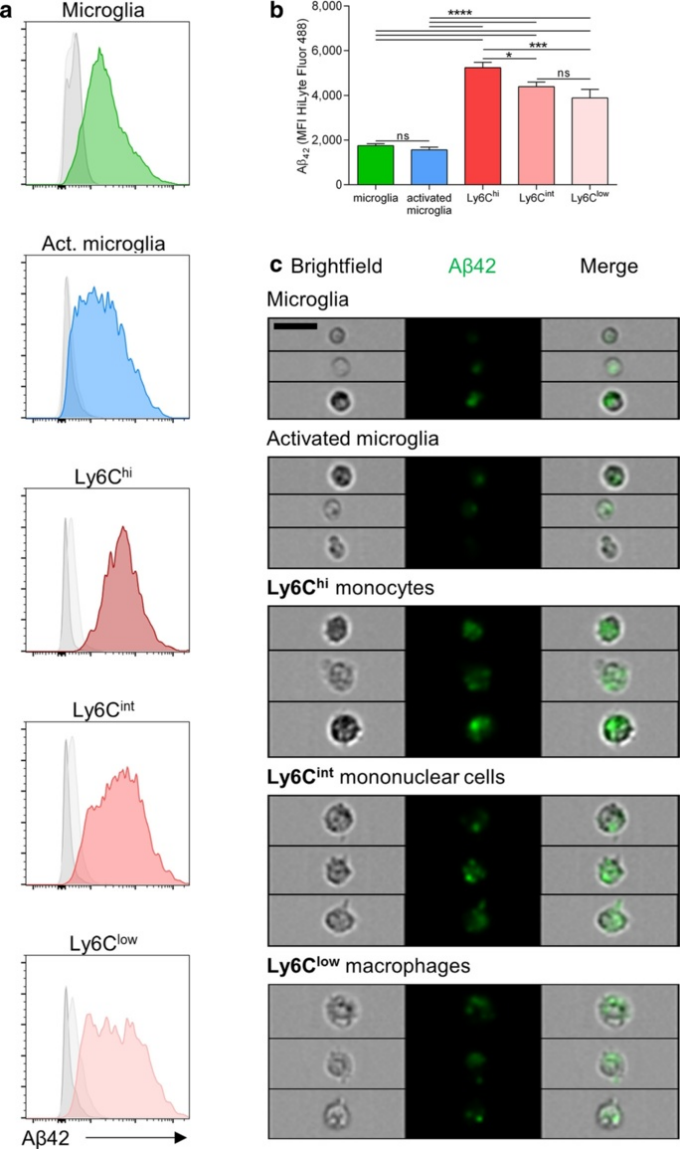
Recruited mononuclear cells are potent Aβ phagocytic cells. **a**–**c** *Ex vivo* phagocytosis assay was performed with mononuclear cells isolated from 5xFAD and C57BL/6 mouse brains and cleared from dead cells by sorting via flow cytometer. **a** Populations were gated as described in Fig. 3a. Representative histograms show the uptake of fluorescence labeled Aβ_42_ peptide by different cell populations. Gray curves show the 4 °C control (*dark gray*) and the Aβ_42_^−^ control (*light gray*) for each population. **b** Bars indicate the median fluorescence intensity (MFI) of each population to express differences in the amount of Aβ_42_ taken up. Data are displayed as mean + SEM (*n* = 4-5). **c** Representative images obtained with FlowSight™ are shown for each population. Scale bar, 20 μm. Significance levels (*p* values) determined by Fisher’s LSD test are indicated. ns, not significant, *p ≤ 0.05, **p ≤ 0.01, ***p ≤ 0.001, ****p ≤ 0.0001

Resident and recruited immune cell subsets display different phagocytic capacity [23]. Here we compared mononuclear cell subpopulations with respect to their ability to specifically phagocytose Aβ_42_ in an *ex vivo* phagocytosis assay. To this end, we freshly isolated brain mononuclear cells and exposed them to Aβ_42_. Resident surveilling microglia (CD45^int^ CD11b^+^), activated microglia (CD45^+^ CD11b^+^) as well as Ly6C^hi^ monocytes, Ly6C^int^ and Ly6C^low^ cells (CD45^hi^ CD11b^hi^ Ly6G^−^ Ly6C^hi^/^int^/^low^) were distinguished by flow cytometric analysis. The low ability of resident microglia to take up Aβ_42_ was reflected in their low median fluorescence intensity (MFI) for Aβ_42_-HiLyte Fluor 488 (1749 ± 88, Fig. 4b, green bar). Upon *T.* *gondii* infection, microglia cells became activated, but their Aβ_42_ uptake remained low (1564 ± 119, Fig. 4b, blue bar). Notably, Ly6C^hi^ monocytes exhibited the highest MFI (5238 ± 239, Fig. 4b, dark red bar) suggesting greater phagocytic capacity. Similarly, also Ly6C^int^ and Ly6C^low^ cells were found to exhibit significantly higher MFI compared to microglia or activated microglia (Ly6C^int^ 4396 ± 204, Ly6C^low^ 3890 ± 387, Fig. 4b, medium and light red bars, *p* < 0.0001). Relative MFIs measured in cells isolated from wildtype mice showed a similar pattern, confirming that Aβ42 uptake was not restricted to 5xFAD cells (Additional file 1: Figure S1).

We further verified that the detected fluorescence resulted from internalized Aβ_42_ rather than from surface-bound signals. Therefore, we analyzed the cells using imaging flow cytometry that allows obtaining images of individual cells. Gating was performed as described in Fig. 3a and representative pictures for each population are shown in Fig. 4c. Microglia and activated microglia populations contained lower numbers of Aβ^+^ cells (Table 1, first column). Consistent with the previous measurements by conventional flow cytometry, fluorescence was low or absent in resting and activated microglia, but all monocyte populations showed an intense signal (Fig. 4c and Additional file 1: Figure S1). Fluorescence was distributed equally across the cells, sometimes with several additional bright spots inside individual cells. This further indicated the uptake of Aβ_42_ by recruited mononuclear cells (CD45^hi^ CD11b^hi^ Ly6G^−^ Ly6C^hi^/^int^/^low^) upon *T.* *gondii* infection. Morphologically, monocytes and the other two monocyte-derived cell subsets were more granular than (activated) microglia, represented by higher side scatter intensities (Fig. 4c and Table 1, last column). We quantified the internalization of Aβ_42_ by calculating the ratio of the fluorescence intensity within the cell to the intensity of the entire cell. Ratios higher than 0 indicate intermediate to high internalization and were found in more than 97 % of all Aβ^+^ cells regardless of the population (Table 1, second column). The mean internalization varied between populations with activated microglia being the lowest and Ly6C^hi^ monocytes being the highest (Table 1, third column).

**Table 1:** Quantification of Aβ_42_ uptake by imaging flow cytometry

Population	Aβ + cells	ratio > 0	Mean ratio	Mean side scatter intensity
Microglia	77.8 ± 2.5 %	99.3 ± 0.2 %	5.0 ± 0.1	1916 ± 85
Activated microglia	71.8 ± 2.3 %	97.1 ± 0.2 %	3.0 ± 0.02	1161 ± 35
Ly6C^hi^	99.4 ± 0.09 %	99.8 ± 0.02 %	5.4 ± 0.06	3574 ± 143
Ly6C^int^	96.1 ± 0.5 %	99.4 ± 0.1 %	5.0 ± 0.1	3376 ± 130
Ly6C^low^	92.8 ± 0.8 %	99.0 ± 0.05 %	4.8 ± 0.08	3107 ± 93

**Fig. 5:**
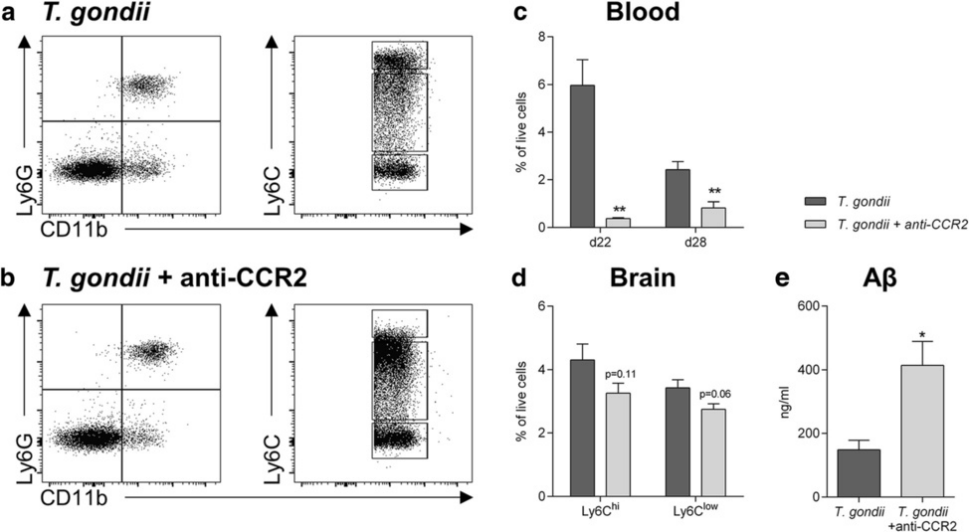
Ablation of CCR2^+^Ly6C^hi^ monocytes increases Aβ accumulation in *T.* *gondii* infected 5xFAD mice. *T.* *gondii* infected 5xFAD mice were treated with the anti-CCR2 monoclonal antibody MC-21 to specifically ablate CCR2^+^Ly6C^hi^ monocytes. **a**, **b** Ly6C^hi^ monocytes in the blood were measured 28 days post infection and after 13 days of anti-CCR2 administration. Representative dot plots picture the gating strategy and the specific ablation of Ly6C^hi^ monocytes in (**b**) anti-CCR2 treated animals compared to (**a**) PBS treated animals. **c**, **d** Ly6C^hi^ monocytes in the blood on d22 and d28 as well as in the brain on d28 were analyzed and their percentage of live cells is displayed. **e** The amount of Aβ_42_ in the brains of 5xFAD mice after *T.* *gondii* infection with and without monocyte ablation was measured by ELISA. Data (*n* = 4 per group) are presented as mean + SEM. Significance levels (*p* values) determined by unpaired Student’s t test are indicated. *p ≤ 0.05, **p ≤ 0.01

To confirm that it is indeed the recruited monocytes and their progeny contributing to plaque removal, we ablated Ly6C^hi^ monocytes using a monoclonal anti-CCR2 antibody. We chose a lower antibody concentration to only reduce monocyte numbers, because the complete elimination would highly increase the susceptibility of infected mice as recently described by us [23]. One week after initiating the ablation, we detected significantly reduced Ly6C^hi^ monocyte levels in the blood (Fig. 5a, b, c). After 2 weeks of anti-CCR2 antibody administration (28 days after infection), Ly6C^hi^ monocyte numbers were still significantly reduced in the blood (Fig. 5b, c). This peripheral depletion led to a trending reduction of Ly6C^hi^ monocytes and Ly6C^low^ monocyte-derived macrophages in the brain on day 28 after *T.* *gondii* infection (Fig. 5d). From the isolated brains, we quantified Aβ_42_ by ELISA. Importantly, diminished Ly6C^hi^ monocyte numbers were associated with an increased total amount of Aβ_42_ in the brain of *T.* *gondii* infected 5xFAD mice (*T.* *gondii* 148 ± 30 ng/ml, *T.* *gondii* + anti-CCR2 414 ± 75 ng/ml, Fig. 5e).

**Fig. 6:**
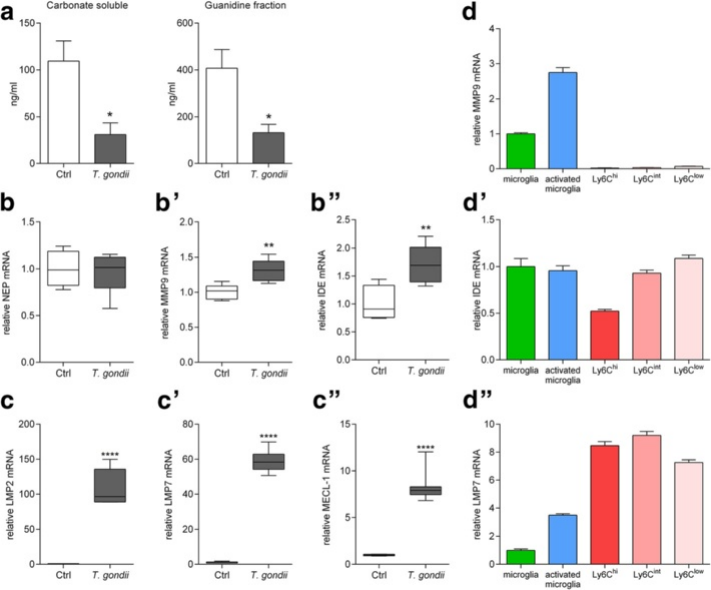
*T.* *gondii* infection reduces small and large Aβ aggregates and enhances mRNA expression of the Aβ degrading enzymes IDE and MMP9 as well as immunoproteasomal subunits. **a** Aβ_42_ in the carbonate soluble (monomeric and small oligomeric Aβ_42_ aggregates) and guanidine soluble fractions (large Aβ_42_ aggregates) of whole-brain homogenates was measured by ELISA. Data are presented as mean + SEM. **b**, **b’**, **b”** Expression of neprilysin (NEP), matrix metalloproteinase 9 (MMP9) and insulysin (IDE) in the brain was measured by RT-PCR in non-infected (*n* = 5) and *T.* *gondii* infected (*n* = 7) 5xFAD mice. Data are presented as fold-change over non-infected 5xFAD mice in box and whisker graphs. **c**, **c’**, **c”** Expression of the immunoproteasome subunits LMP2, LMP7 and MECL-1 in the brain was measured by RT-PCR in non-infected (*n* = 5) and *T.* *gondii* infected (*n* = 7) 5xFAD mice. Data are presented as fold change over non-infected 5xFAD mice in box and whisker graphs. **d**, **d’**, **d”** Expression of the MMP9, IDE and LMP7 by innate immune cells isolated and sorted from the brains of non-infected and *T.* *gondii* infected C57BL/6 mice was measured by RT-PCR. Each sample consists of pooled cells from six animals and was measured in triplicates. Significance levels (*p* values) determined by unpaired Student’s t test or Fisher’s LSD test are indicated. *p ≤ 0.05, **p ≤ 0.01, ****p ≤ 0.0001

While immunohistological methods (Fig. 1) can only identify Aβ plaques, ELISA measurement allows the quantification of total Aβ. Using a sequential protocol, we separated monomeric and small oligomeric Aβ (carbonate-soluble) from larger aggregates (guanidine-soluble) in whole-brain homogenates. Next, we quantified the amount of Aβ_42_ and found that it was significantly reduced in both fractions (carbonate soluble: control 109.3 ± 21.6 ng/ml, *T.* *gondii* 31.1 ± 12.5 ng/ml, *p* < 0.05; guanidine fraction: control 406.9 ± 79.9 ng/ml, *T.* *gondii* 132.3 ± 35.2 ng/ml, *p* < 0.05, Fig. 6a).

### Recruited mononuclear cells increase proteolytic clearance of Aβ

Along with uptake of Aβ, its proteolytic processing is equally important. Therefore, we measured the expression of three Aβ-degrading enzymes in the brain, namely insulin-degrading enzyme (*IDE*), neprilysin (*NEP*), and matrix metalloproteinase 9 (*MMP9*), using RT-PCR, and we observed a significant increase in the expression of *MMP9* and *IDE* mRNA following infection with *T.* *gondii* (*MMP9*: 1.3 ± 0.1 fold-change over 5xFAD controls, *p* < 0.01, Fig. 6b’; *IDE*: 1.7 ± 0.1 fold-change over 5xFAD controls, *p* < 0.01, Fig. 6b”). The expression of *NEP* remained unaltered (Fig. 6b, 0.94 ± 0.1 fold-change over 5xFAD controls, *p* > 0.6).

Another suggested degradation pathway is the ubiquitin proteasome system (UPS). The UPS is the major intracellular protein degradation machinery and includes the proteolytic subunits β1i/LMP2, β2i/MECL-1, and *β*5i/LMP7. We detected a strong increase of mRNA expression for the immunoproteasome subunits *β1i/LMP2*, *β2i/MECL-1*, and *β5i/LMP7* in whole-brain RNA from *T.* *gondii* infected 5xFAD mice (*β1i/LMP2*: 108 ± 9 fold-change over 5xFAD controls, *p* < 0.0001, Fig. 6c; *β2i/MECL-1*: 8 ± 0.7 fold-change over 5xFAD controls, *p* < 0.0001, Fig. 6c’; *β5i/LMP7*: 59 ± 2 fold-change over 5xFAD controls, *p* < 0.0001, Fig. 6c”). Interestingly, detailed investigation of brain resident as well as recruited immune cells in *T.* *gondii* infected mice revealed a prominent mRNA expression of *β5i/LMP7* in recruited mononuclear cells (Fig. 6d”) in contrast to other Aβ degrading mechanisms. *MMP9* mRNA was upregulated in activated compared to surveilling microglia, but almost undetectable in recruited mononuclear cells (Fig. 6d). *IDE* mRNA was similarly expressed across all innate immune cell populations we investigated (Fig. 6d’). Notably, *LMP7* mRNA expression was more than twofold higher in the sorted recruited mononuclear cells in the brain when compared to surveilling or activated microglia, indicating a new mechanism of Aβ clearance. Of note, in the chronic stage of infection the intracellular parasites form cysts within neurons hiding from the immune system, thus the analyzed cells were not directly infected with tachyzoites.

**Fig. 7:**
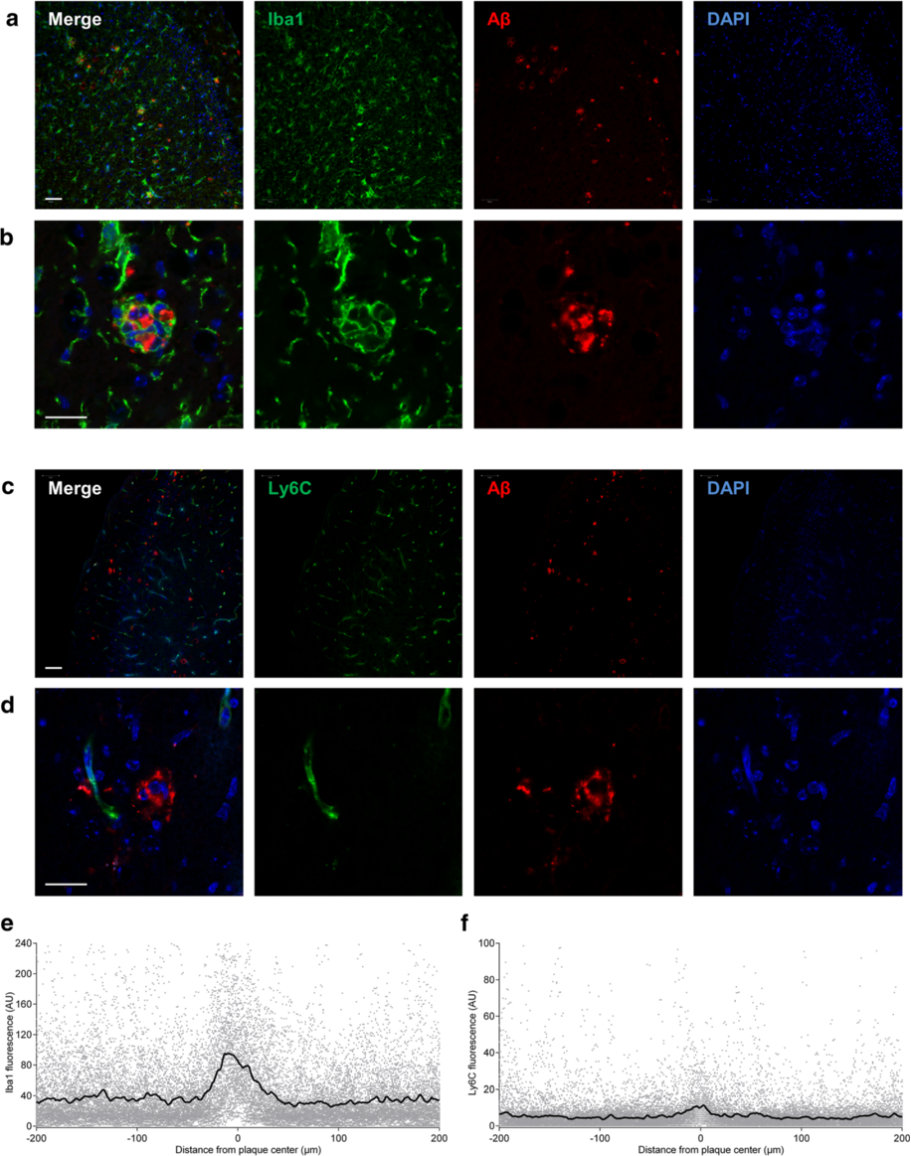
Microglia but not Ly6C^hi^ monocytes are located in the vicinity of Aβ plaques. **a**-**d** show immunolabeled coronal sections from *T.* *gondii* infected 5xFAD mice. **a**, **b** Co-labeling of Iba1 (microglia) and Aβ (plaques) reveals close interaction of microglia with plaques. **a** Low magnification (20x) overview of the cortex. Scale bar, 200 μm. **b** Representative cortical plaque surrounded by microglial processes. 63x magnification, scale bar, 20 μm. **c**, **d** In sections co-labeled for Ly6C (Ly6C^hi^ monocytes) and Aβ (plaques), Ly6C^hi^ monocytes were not located in the direct vicinity of plaques. **c** Low magnification (20x) overview of the cortex. Scale bar, 200 μm. **d** Representative cortical plaque with a nearby Ly6C^+^ blood vessel but no associated Ly6C^hi^ monocytes. 63x magnification, scale bar, 20 μm. **e**, **f** The sections were analyzed to quantify the fluorescence intensity of (**e**) Iba1 and (**f**) Ly6C in the direct environment of plaques. The black line represents the average at a given distance. AU, arbitrary units

Furthermore, we investigated which cell types are located around the Aβ plaques in the cortex of *T.* *gondii* infected 5xFAD mice by performing immunofluorescence stainings against Iba1, Ly6C, and Aβ. Due to their general distribution in the parenchyma, Iba1^+^ microglia and monocyte-derived macrophages were closely associated with Aβ plaques (Fig. 7a, b). Interestingly, Ly6C^+^ cells were not located directly in the vicinity of Aβ plaques (Fig. 7c, d). As to that, it is important not to confuse Ly6C^+^ monocytes with Ly6C expressing endothelial cells [75], which can be recognized by their elongated shape. This qualitative finding was confirmed by quantification of fluorescence intensity around plaques (Fig. 7e, f).

Taken together, our results show that Ly6C^hi^, Ly6C^int^, and Ly6C^low^ mononuclear cells are highly capable of removing soluble Aβ peptides. While Ly6C^hi^ monocytes contribute significantly to this clearance, they cannot be found in the direct vicinity of established plaques and thus, may rather lower the general amount of Aβ to prevent higher molecular weight Aβ aggregates and plaque formation.

## Discussion

In the present study, we investigated the effect of the commonly persisting cerebral *Toxoplasma* infection and resulting CNS inflammation on Aβ plaque formation in a murine model of AD. In the following, we will first discuss the etiologic connection between *T.* *gondii* and AD and how our results contribute to the understanding of this connection. Second, we will address the individual results and evaluate what we learned from this experimental setup with respect to a possible treatment of AD.

### Etiologic connection between *T.* *gondii* and AD

Aβ and hyperphosphorylated tau form disease promoting aggregates in AD that trigger chronic cerebral inflammatory processes [76, 77]. Further modulation of the chronic inflammation may occur following several infectious diseases, which are known to induce inflammatory cascades in the CNS. It is well established that certain pathogenic components modulate the course of disease in murine β-amyloidosis models [78]. Although the impact of particular infections during the pathogenesis of AD has been discussed, the underlying mechanisms are especially challenging to untangle (reviewed in [38]). In general, previous studies have found chronic or latent infections to be associated with an increased risk for AD (reviewed in [38]), although evidence regarding *T.* *gondii* specifically is inconclusive [54, 55]. On the other hand, a recent study using a mouse model of cerebral β-amyloidosis, *T.* *gondii* infection has been associated with a reduced risk of developing AD-like pathology [58]. Our study provides further evidence for this notion, as we detected a significant reduction in the number and volume of β-amyloid plaques in *T.* *gondii* infected compared to non-infected 5xFAD mice.

Despite this observed reduction in plaque deposition, one should be cautious in concluding that *T.* *gondii* infection may protect against developing AD. In fact, while evidence regarding actual disease risk is sparse and conflicting [54, 55], latent toxoplasmosis in healthy individuals has recently been associated with subtle reductions of cognitive performance in various tasks [79–81]. Directly translating this finding to the situation of human AD patients is rather difficult, considering the limitations of the applied experimental model. Low dose infection of mice with the parasites is a broadly used model to mimic human chronic *Toxoplasma* infection [18, 23, 44, 46, 49, 82–85], although the immune cell recruitment in mice is most likely more pronounced than during latent chronic infection of humans. Considering that we propose the recruited immune cells as major mediators of the beneficial effect, it is unclear which effects are applicable to the human CNS.

### Insights regarding the treatment of AD

As mentioned above, we measured decreased levels of Aβ plaques in the brains of *T.* *gondii* infected 5xFAD mice and the remaining plaques were also smaller in volume. These findings are consistent with the aforementioned study by Jung and colleagues, where they further described an improved performance in behavioral tests compared to non-infected transgenic mice [58].

We performed a more detailed investigation of the Aβ load and detected that the general reduction of Aβ plaques was paralleled by a decrease in small soluble Aβ species. Because higher levels of soluble Aβ has previously been linked to decreased cognitive performance [86, 87], this finding could suggest that our experimental setup at least partially protects from cognitive decline.

Controlling the infection with *T.* *gondii* requires the collaboration of innate and adaptive immunity [48]. Even though there are inflammatory diseases of the CNS which involve adaptive immune cells, such as multiple sclerosis, the inflammation observed in AD seems to be restricted to innate immune cells [88]. Thus, we focused our further analysis on the contribution of the innate compartment.

The detailed analysis of infiltrating immune cells confirmed the strong recruitment of innate immune cells, particularly CD45^hi^ CD11b^hi^ myeloid cells, to the brains of *T.* *gondii* infected 5xFAD mice, similar to that seen in wildtype mice. It has been described previously by our group that in mice chronically infected with *T.* *gondii*, Ly6C^hi^ monocytes migrate to the CNS and further differentiate into Ly6C^int^ mononuclear cells and Ly6C^low^ macrophages in order to carry out specific tasks in host defense, such as cytokine production and Fc receptor mediated cellular phagocytosis [18, 23].

We analyzed the contribution of these myeloid-derived mononuclear cell subsets in the process of accumulating Aβ plaques that, according to the widely accepted view on AD pathophysiology, ultimately promote neurodegeneration.

*Ex vivo* phagocytosis assay most likely by an antibody-independent mechanism revealed that all recruited mononuclear subpopulations were able to take up significantly more Aβ_42_ than microglia from non-infected or activated microglia from *T.* *gondii* infected 5xFAD mice. We found that Ly6C^hi^ monocytes displayed an even higher uptake compared to Ly6C^low^ cells. Comparing the ability to take up Aβ_42_ with the ability to take up latex spheres as previously published [23], we noted a difference with respect to the Ly6C^hi^ and Ly6C^int^ subsets. While their uptake of latex spheres is very low [23], they displayed a prominent uptake of Aβ_42_. Even though our results are against the general view that Ly6C^low^ cells are the most macrophage-like subset, other publications have attributed phagocytic activity to Ly6C^+^ monocytes against parasites [89, 90]. Thus, we conclude that uptake of latex beads and Aβ_42_ is mediated by different mechanisms with diverse appearance in Ly6C^hi^ monocytes and Ly6C^low^ macrophages.

While neuroinflammation has been conventionally reported to be detrimental and associated with several neurological diseases [91–93], emerging research promotes a more differentiated view on the roles of recruited immune cells in homeostatic and repair mechanisms [12, 13, 27, 94, 95]. Consistent with this concept, there are a growing number of reports indicating the beneficial effect of recruited immune cells in AD and vascular amyloidosis [28–30, 96–98].

Performing the *ex vivo* phagocytosis assay with cells obtained from both wildtype and 5xFAD mice, we also observed that the relative contributions were independent of the genotype, despite absolute values being different. As these differences were most likely caused by the different quantification methods, we conclude that wildtype cells are potent Aβ clearing cells as well. Importantly, this finding suggests that cells probably do not have to be pre-exposed to Aβ to efficiently phagocytose Aβ in a possible treatment strategy. It is somehow challenging that human macrophages were found to be ineffective at Aβ phagocytosis when derived from AD patients [99]. Nevertheless, modulating the route of entry may provide a tool to skew recruited monocytes towards an inflammation resolving phenotype [22] and the capacity of these manipulated monocytes to remove Aβ remains to be investigated, as two studies have found that the replacement of brain resident microglia with peripheral myeloid cells does not reduce the Aβ burden [100, 101]. Both studies used a similar approach to replace microglia with peripheral cells, i. e. depletion of brain resident CD11b-expressing cells during a 10 to 14 days intracerebral ganciclovir treatment of CD11b-HSVTK (herpes simplex virus thymidine kinase) transgenic mice [100, 101]. This treatment leads to a one-time replacement with bone marrow-derived myeloid cells, as opposed to the continuous influx observed in our model of chronic cerebral *T.* *gondii* infection. Additionally, Prokop and colleagues point out the lack of an activating stimulus in their model, which would be able to induce the uptake of Aβ by myeloid cells [100]. Even though this lack of stimulation is resolved in our experimental model, finding appropriate stimuli to manipulate the cells is a complex task, as we have to keep in mind that beneficial and detrimental effects of monocytes and macrophages can occur at the same time [102].

The increased amount of Aβ_42_ detected following CCR2^hi^ Ly6C^hi^ monocyte ablation in infected 5xFAD mice points to a causal role of these cells to Aβ clearance. Our findings are supported by a report from Naert and Rivest, who have linked the lack of Ly6C^hi^ (CX3CR1^low^ CCR2^+^ Gr1^+^) monocytes to cognitive decline in APP_Swe_/PS1 mice [65]. This hypothesis is further strengthened by two very recent studies where myeloid cell recruitment to the CNS was correlated with Aβ plaque reduction [30, 103]. In a very recently published study, Baruch and colleagues proposed a novel treatment strategy to target AD via programmed death-1 (PD-1) inhibition and thereby increasing the recruitment of Ly6C^hi^ monocytes to the CNS in an IFN-γ dependent manner [104]. The proposed mechanisms included enhanced cellular uptake and degradation. Furthermore, Savage et al. detected phagocytic cells directly associated with plaques, and the CD45^hi^ status of these cells suggested their myeloid origin [105]. Only short-term recruitment of monocytes did not alter plaque deposition as seen in a mouse model of traumatic brain injury [106].

Having confirmed Ly6C^hi^ monocytes as key contributors to Aβ removal in our model, we were interested if they migrate into the parenchyma to “attack” Aβ plaques like previous reports have shown [28, 103, 107]. However, CCR2^+^ Ly6C^+^ monocytes were not located in the vicinity of plaques in our experiments. Therefore, we propose that the low plaque burden in the applied experimental model is due to Ly6C^hi^ monocytes’ increased capacity to remove soluble Aβ rather than due to direct removal of established plaques. In addition, monocyte-derived Ly6C^low^ macrophages upregulate F4/80 and Iba1, and can be located adjacent to the plaques, similarly to resident microglia.

It has to be carefully investigated, at which stages of disease the recruitment of monocytes and subsequent removal of Aβ is beneficial and can delay the onset of disease, and at which stages the cascade triggered by Aβ is already on its way and additional cell recruitment potentially worsens neuroinflammation [108]. Our data from old animals provides evidence that the ability of freshly recruited immune cells to remove Aβ persists at later stages of experimental amyloidosis.

Searching for a mechanism mediating Aβ uptake, we analyzed the expression of cell surface markers related to phagocytosis on CD11b^hi^ Ly6G^−^ myeloid-derived cells. The measurements revealed intermediate levels of TREM2, CD36 and SCARA1 on Ly6C^hi^ monocytes and high levels on Ly6C^low^ monocyte-derived macrophages. Recent reports of a correlation between genetic TREM2 mutation and AD [109, 110], along with experiments pointing out the anti-inflammatory and phagocytosis-enhancing role of TREM2, have drawn the attention towards this molecule [111–114]. We detected that monocyte-derived Ly6C^low^ macrophages expressed high levels of TREM2, in contrast to Ly6C^hi^ monocytes. This result underlines that, besides TREM2, other factors may determine the capacity of immune cells such as monocytes to phagocytose Aβ. Several studies have suggested that CD36 expression is associated with Aβ uptake [76, 115, 116], consistent with the CD36 expression of Ly6C^low^ monocyte-derived macrophages. Moreover, the lower expression of CD36 detected on Ly6C^hi^ monocytes may be beneficial because of less harmful pro-inflammatory CD36-Aβ interaction [117–120]. Frenkel and colleagues had shown that SCARA1 (and not CD36) mediates phagocytosis of Aβ [118]. However, similar to TREM2 and CD36, SCARA1 expression was not a reliable predictor of the Aβ phagocytic capacity of each myeloid-derived mononuclear cell subset in our model.

Recent research highlights the importance of proteolytic Aβ degradation. Several enzymes are known to digest Aβ, including MMP9 and IDE, but the contribution of each enzyme is crucial. Removal of only one can result in significantly increased cerebral Aβ levels [121], and overexpression leads to decreased Aβ loads [121–123]. We found both *MMP9* and *IDE* upregulated significantly upon *T.* *gondii* infection in 5xFAD mice.

MMP9 is one of several matrix metalloproteinases that have been implicated in Aβ degradation and administration of an MMP inhibitor resulted in increased Aβ loads [121]. Activation of MMPs has to be regarded carefully as well, as a recent study has shown that Aβ increases the permeability of the BCSFB by activation of MMPs [124]. Even though Brkic and colleagues found the biggest changes for MMP3 expression, the contribution of other MMPs cannot be ruled out. IDE hydrolytically cleaves Aβ [125], and the significantly increased expression of *IDE*, particularly in conjunction with the simultaneously increased *MMP9* expression, therefore most likely promoted the enhanced degradation of Aβ in *T.* *gondii* infected 5xFAD mice, when compared to non-infected controls. Upregulation of *IDE* may be a compensatory mechanism, since insulin has been shown to stimulate the growth of *T.* *gondii* in vitro [126], and increased Aβ degradation could be a beneficial secondary effect.

In addition, intracellular control of protein homeostasis is mediated by the ubiquitin-proteasome system, whereby proteasomes represent the proteolytically active part. Dysfunction of the UPS has been shown to be an early event in Alzheimer’s disease, suggesting that proteasomes may be unable to properly degrade ubiquitin-tagged proteins [127]. Immunoproteasomes are specific proteasome isoforms that have incorporated the immunosubunits β1i/LMP2, β2i/MECL-1, and β5i/LMP7. Previous data point to an important role of immunoproteasomes in the rapid degradation of oxidant-damaged proteins, thus expression of immunoproteasomes may be beneficial in Aβ clearance [128]. Indeed, recently published data indicate that reactive glia exhibit induced immunoproteasome expression and activation in the cortex of a plaque pathology mouse model [129]. On the other hand, β5i/LMP7-deficiency has been shown to result in attenuation of lymphocytic choriomeningitis virus (LCMV)-induced meningitis [130, 131]. Notably, our data presented here demonstrate the most prominent *β5i/LMP7* expression in recruited mononuclear cell subsets, indicating that high immunoproteasome expression in monocytes and their progeny are associated with their enhanced capacity in Aβ degradation and suggesting a novel mechanism of Aβ elimination. However, the exact engagement of the UPS in the mentioned processes requires detailed investigation in forthcoming experiments.

## Conclusions

Taken together, our results demonstrate that mononuclear cell recruitment to the brain upon chronic *T.* *gondii* infection leads to reduced plaque burden by promoting phagocytosis of soluble Aβ_42_ and enhanced proteolytic degradation. This aspect is especially critical in a situation where resident microglia are dysfunctional and fail to control amyloid plaque deposition [67, 132]. Chronic *T.* *gondii* infection acts as a strong immunological stimulus, possibly even overcoming the impaired phagocytic capacities of monocytes/macrophages as observed in AD patients [99]. In the light of a future treatment option, a recent study by Neal and Knoll presented data from mice showing that infection with *T.* *gondii* protects from bacterial infection with *Listeria monocytogenes* by recruitment of Ly6C^hi^ monocytes. Further experiments revealed that only application of a component of *T.* *gondii* is sufficient to mediate the resistance [133]. This method of recruiting monocytes may be interesting to consider, as our results suggest a promising candidate mechanism for the protective effect, namely increased phagocytosis and degradation of Aβ.

## Additional file

Additional file 1: Figure S1.
*Ex vivo* Aβ_42_ uptake by cells from wildtype C57BL/6 mice. *Ex vivo* phagocytosis assay was performed with mononuclear cells isolated from C57BL/6 mouse brains. Populations were gated as described in Fig. 3a and Aβ_42_ uptake was quantified using imaging flow cytometry. Bars indicate the median fluorescence intensity (MFI) of each population and data are displayed as mean + SEM (*n* = 5). Significance levels (*p* values) determined by Fisher’s LSD test are indicated. ns, not significant, **p* ≤ 0.05, ***p* ≤ 0.01, *****p* ≤ 0.0001. (TIF 144 kb)

## References

[1] Reitz C, Brayne C, Mayeux R (2011). Epidemiology of Alzheimer disease. Nat Rev Neurol.

[2] Hardy J, Selkoe DJ (2002). The amyloid hypothesis of Alzheimer’s disease: progress and problems on the road to therapeutics. Science.

[3] Selkoe DJ (2001). Alzheimer’s disease: genes, proteins, and therapy. Physiol Rev.

[4] Haass C, Kaether C, Thinakaran G, Sisodia S (2012). Trafficking and proteolytic processing of APP. Cold Spring Harb Perspect Med.

[5] Masters CL, Selkoe DJ (2012). Biochemistry of amyloid β-protein and amyloid deposits in Alzheimer disease. Cold Spring Harb Perspect Med.

[6] Mawuenyega KG, Sigurdson W, Ovod V (2010). Decreased clearance of CNS beta-amyloid in Alzheimer’s disease. Science.

[7] Pahnke J, Langer O, Krohn M. Alzheimer’s and ABC transporters - new opportunities for diagnostics and treatment. Neurobiol Dis. 2014. 1–7. doi:10.1016/j.nbd.2014.04.001 10.1016/j.nbd.2014.04.001 PMC4199932 24746857

[8] Pahnke J, Walker LC, Scheffler K, Krohn M (2009). Alzheimer’s disease and blood–brain barrier function-Why have anti-beta-amyloid therapies failed to prevent dementia progression?. Neurosci Biobehav Rev.

[9] Elali A, Rivest S (2013). The role of ABCB1 and ABCA1 in beta-amyloid clearance at the neurovascular unit in Alzheimer’s disease. Front Physiol.

[10] Abuznait AH, Kaddoumi A (2012). Role of ABC transporters in the pathogenesis of Alzheimer’s disease. ACS Chem Neurosci.

[11] Johnston H, Boutin H, Allan SM (2011). Assessing the contribution of inflammation in models of Alzheimer’s disease. Biochem Soc Trans.

[12] Pahnke J, Fröhlich C, Krohn M (2013). Impaired mitochondrial energy production and ABC transporter function-A crucial interconnection in dementing proteopathies of the brain. Mech Ageing Dev.

[13] Fröhlich C, Paarmann K, Steffen J (2013). Genomic background-related activation of microglia and reduced β-amyloidosis in a mouse model of Alzheimer’s disease. Eur J Microbiol Immunol (Bp).

[14] Ritchie K, Lovestone S (2002). The dementias. Lancet.

[15] Nguyen MD, Julien J-P, Rivest S (2002). Innate immunity: the missing link in neuroprotection and neurodegeneration?. Nat Rev Neurosci.

[16] Simard AR, Rivest S (2006). Neuroprotective properties of the innate immune system and bone marrow stem cells in Alzheimer’s disease. Mol Psychiatry.

[17] Shechter R, Schwartz M (2013). Harnessing monocyte-derived macrophages to control central nervous system pathologies: no longer “if” but “how”. J Pathol.

[18] Möhle L, Parlog A, Pahnke J, Dunay IR (2014). Spinal cord pathology in chronic experimental Toxoplasma gondii infection. Eur J Microbiol Immunol (Bp).

[19] Ginhoux F, Greter M, Leboeuf M (2010). Fate mapping analysis reveals that adult microglia derive from primitive macrophages. Science.

[20] Ajami B, Bennett JL, Krieger C (2011). Infiltrating monocytes trigger EAE progression, but do not contribute to the resident microglia pool. Nat Neurosci.

[21] Baruch K, Kertser A, Porat Z, Schwartz M (2015). Cerebral nitric oxide represses choroid plexus NFκB-dependent gateway activity for leukocyte trafficking. EMBO J.

[22] Schwartz M, Baruch K (2014). The resolution of neuroinflammation in neurodegeneration: leukocyte recruitment via the choroid plexus. EMBO J.

[23] Biswas A, Bruder D, Wolf SA (2015). Ly6Chigh Monocytes Control Cerebral Toxoplasmosis. J Immunol.

[24] London A, Benhar I, Mattapallil MJ (2013). Functional macrophage heterogeneity in a mouse model of autoimmune central nervous system pathology. J Immunol.

[25] Kunis G, Baruch K, Miller O, Schwartz M (2015). Immunization with a Myelin-Derived Antigen Activates the Brain’s Choroid Plexus for Recruitment of Immunoregulatory Cells to the CNS and Attenuates Disease Progression in a Mouse Model of ALS. J Neurosci.

[26] Prokop S, Miller KR, Heppner FL (2013). Microglia actions in Alzheimer’s disease. Acta Neuropathol.

[27] Shechter R, London A, Varol C (2009). Infiltrating blood-derived macrophages are vital cells playing an anti-inflammatory role in recovery from spinal cord injury in mice. PLoS Med.

[28] Simard AR, Soulet D, Gowing G (2006). Bone marrow-derived microglia play a critical role in restricting senile plaque formation in Alzheimer’s disease. Neuron.

[29] Naert G, Rivest S (2012). Hematopoietic CC-chemokine receptor 2 (CCR2) competent cells are protective for the cognitive impairments and amyloid pathology in a transgenic mouse model of Alzheimer’s disease. Mol Med.

[30] Koronyo Y, Salumbides BC, Sheyn J (2015). Therapeutic effects of glatiramer acetate and grafted CD115+ monocytes in a mouse model of Alzheimer’s disease. Brain.

[31] Jurgens HA, Amancherla K, Johnson RW (2012). Influenza infection induces neuroinflammation, alters hippocampal neuron morphology, and impairs cognition in adult mice. J Neurosci.

[32] McManus RM, Higgins SC, Mills KHG, Lynch MA (2014). Respiratory infection promotes T cell infiltration and amyloid-β deposition in APP/PS1 mice. Neurobiol Aging.

[33] Cunningham C (2013). Microglia and neurodegeneration: the role of systemic inflammation. Glia.

[34] Holmes C (2013). Review: systemic inflammation and Alzheimer’s disease. Neuropathol Appl Neurobiol.

[35] Krstic D, Madhusudan A, Doehner J (2012). Systemic immune challenges trigger and drive Alzheimer-like neuropathology in mice. J Neuroinflammation.

[36] Harris SA, Harris EA (2015). Herpes Simplex Virus Type 1 and Other Pathogens are Key Causative Factors in Sporadic Alzheimer’s Disease. J Alzheimers Dis.

[37] Carter C (2011). Alzheimer’s Disease: APP, Gamma Secretase, APOE, CLU, CR1, PICALM, ABCA7, BIN1, CD2AP, CD33, EPHA1, and MS4A2, and Their Relationships with Herpes Simplex, C. Pneumoniae, Other Suspect Pathogens, and the Immune System. Int J Alzheimers Dis.

[38] Miklossy J (2011). Emerging roles of pathogens in Alzheimer disease. Expert Rev Mol Med.

[39] Gavazzi G, Krause K-H (2002). Ageing and infection. Lancet Infect Dis.

[40] Montoya JG, Liesenfeld O (2004). Toxoplasmosis. Lancet.

[41] Pinchinat S, Cebrián-Cuenca AM, Bricout H, Johnson RW (2013). Similar herpes zoster incidence across Europe: results from a systematic literature review. BMC Infect Dis.

[42] Colugnati FB, Staras SS, Dollard SC, Cannon MJ (2007). Incidence of cytomegalovirus infection among the general population and pregnant women in the United States. BMC Infect Dis.

[43] Jones JL, Kruszon-Moran D, Wilson M (2001). Toxoplasma gondii infection in the United States: seroprevalence and risk factors. Am J Epidemiol.

[44] Hermes G, Ajioka JW, Kelly KA (2008). Neurological and behavioral abnormalities, ventricular dilatation, altered cellular functions, inflammation, and neuronal injury in brains of mice due to common, persistent, parasitic infection. J Neuroinflammation.

[45] Ingram WM, Goodrich LM, Robey EA, Eisen MB (2013). Mice infected with low-virulence strains of Toxoplasma gondii lose their innate aversion to cat urine, even after extensive parasite clearance. PLoS One.

[46] Parlog A, Harsan L-A, Zagrebelsky M (2014). Chronic murine toxoplasmosis is defined by subtle changes in neuronal connectivity. Dis Model Mech.

[47] Parlog A, Schlüter D, Dunay IR. Toxoplasma gondii induced neuronal alterations. Parasite Immunol. 2014. doi:10.1111/pim.12157 10.1111/pim.12157 25376390

[48] Dupont CD, Christian DA, Hunter CA. Immune response and immunopathology during toxoplasmosis. Semin Immunopathol. 2012. doi: 10.1007/s00281-012-0339-3 10.1007/s00281-012-0339-3 PMC3498595 22955326

[49] Blanchard N, Dunay IR, Schlüter D (2015). Persistence of Toxoplasma gondii in the central nervous system: a fine-tuned balance between the parasite, the brain and the immune system. Parasite Immunol.

[50] Gordon S, Taylor PR (2005). Monocyte and macrophage heterogeneity. Nat Rev Immunol.

[51] Grainger JR, Wohlfert EA, Fuss IJ (2013). Inflammatory monocytes regulate pathologic responses to commensals during acute gastrointestinal infection. Nat Med.

[52] Dunay IR, Damatta RA, Fux B (2008). Gr1(+) inflammatory monocytes are required for mucosal resistance to the pathogen Toxoplasma gondii. Immunity.

[53] Karlmark KR, Tacke F, Dunay IR (2012). Monocytes in health and disease — Minireview. Eur J Microbiol Immunol.

[54] Kusbeci OY, Miman O, Yaman M (2011). Could Toxoplasma gondii have any role in Alzheimer disease?. Alzheimer Dis Assoc Disord.

[55] Perry CE, Gale SD, Erickson L (2015). Seroprevalence and Serointensity of Latent Toxoplasma gondii in a Sample of Elderly Adults With and Without Alzheimer Disease. Alzheimer Dis Assoc Disord.

[56] Rozenfeld C, Martinez R (2003). Soluble factors released by Toxoplasma gondii-infected astrocytes down-modulate nitric oxide production by gamma interferon-activated microglia and prevent neuronal degeneration. Infect Immun.

[57] Rozenfeld C, Martinez R, Seabra S (2005). Toxoplasma gondii Prevents Neuron Degeneration by Interferon-γ-Activated Microglia in a Mechanism Involving Inhibition of Inducible Nitric Oxide Synthase and Transforming Growth Factor-β1 Production by Infected Microglia. Am J Pathol.

[58] Jung B-K, Pyo K-H, Shin KY (2012). Toxoplasma gondii infection in the brain inhibits neuronal degeneration and learning and memory impairments in a murine model of Alzheimer’s disease. PLoS One.

[59] Oakley H, Cole SL, Logan S (2006). Intraneuronal beta-amyloid aggregates, neurodegeneration, and neuron loss in transgenic mice with five familial Alzheimer’s disease mutations: potential factors in amyloid plaque formation. J Neurosci.

[60] Teipel SJ, Buchert R, Thome J (2011). Development of Alzheimer-disease neuroimaging-biomarkers using mouse models with amyloid-precursor protein-transgene expression. Prog Neurobiol.

[61] Chin J. Selecting a Mouse Model of Alzheimer’s Disease. Methods Mol Biol. 2011. doi:10.1007/978-1-60761-744-0 10.1007/978-1-60761-744-0_13 20967591

[62] Hofrichter J, Krohn M, Schumacher T (2013). Reduced Alzheimer’s disease pathology by St. John's Wort treatment is independent of hyperforin and facilitated by ABCC1 and microglia activation in mice. Curr Alzheimer Res.

[63] Schumacher T, Krohn M, Hofrichter J (2012). ABC transporters B1, C1 and G2 differentially regulate neuroregeneration in mice. PLoS One.

[64] Scheffler K, Krohn M, Dunkelmann T (2012). Mitochondrial DNA polymorphisms specifically modify cerebral β-amyloid proteostasis. Acta Neuropathol.

[65] Schmidt A, Pahnke J (2012). Efficient near-infrared in vivo imaging of amyoid-β deposits in Alzheimer’s disease mouse models. J Alzheimers Dis.

[66] Krohn M, Lange C, Hofrichter J (2011). Cerebral amyloid-β proteostasis is regulated by the membrane transport protein ABCC1 in mice. J Clin Invest.

[67] Scheffler K, Stenzel J, Krohn M (2011). Determination of spatial and temporal distribution of microglia by 230 nm-high-resolution, high-throughput automated analysis reveals different amyloid plaque populations in an APP/PS1 mouse model of Alzheimer’s disease. Curr Alzheimer Res.

[68] Bereswill S, Kühl AA, Alutis M (2014). The impact of Toll-like-receptor-9 on intestinal microbiota composition and extra-intestinal sequelae in experimental Toxoplasma gondii induced ileitis. Gut Pathog.

[69] Rubio-Perez JM, Morillas-Ruiz JM (2012). A review: inflammatory process in Alzheimer’s disease, role of cytokines. Sci. World J..

[70] Perry VH, Nicoll JAR, Holmes C (2010). Microglia in neurodegenerative disease. Nat Rev Neurol.

[71] Fischer HG, Bonifas U, Reichmann G (2000). Phenotype and functions of brain dendritic cells emerging during chronic infection of mice with Toxoplasma gondii. J Immunol.

[72] Clark RT, Nance JP, Noor S, Wilson EH (2011). T-cell production of matrix metalloproteinases and inhibition of parasite clearance by TIMP-1 during chronic Toxoplasma infection in the brain. ASN Neuro.

[73] Schlüter D, Hein A, Dörries R, Deckert-Schlüter M (1995). Different subsets of T cells in conjunction with natural killer cells, macrophages, and activated microglia participate in the intracerebral immune response to Toxoplasma gondii in athymic nude and immunocompetent rats. Am J Pathol.

[74] Naert G, Rivest S (2012). Age-related changes in synaptic markers and monocyte subsets link the cognitive decline of APP(Swe)/PS1 mice. Front Cell Neurosci.

[75] Jutila MA, Kroese FG, Jutila KL (1988). Ly-6C is a monocyte/macrophage and endothelial cell differentiation antigen regulated by interferon-gamma. Eur J Immunol.

[76] Hickman SE, Allison EK, El Khoury J (2008). Microglial dysfunction and defective beta-amyloid clearance pathways in aging Alzheimer’s disease mice. J Neurosci.

[77] Meda L, Cassatella MA, Szendrei GI (1995). Activation of microglial cells by beta-amyloid protein and interferon-gamma. Nature.

[78] Kahn MS, Kranjac D, Alonzo C (2012). Prolonged elevation in hippocampal Aβ and cognitive deficits following repeated endotoxin exposure in the mouse. Behav Brain Res.

[79] Gajewski PD, Falkenstein M, Hengstler JG, Golka K (2014). Toxoplasma gondii impairs memory in infected seniors. Brain Behav Immun.

[80] Gale SD, Erickson LD, Berrett A, et al. Infectious Disease Burden and Cognitive Function in Young to Middle-Aged Adults. Brain Behav Immun. 2015. doi:10.1016/j.bbi.2015.10.014 10.1016/j.bbi.2015.10.014 26598104

[81] Gale SD, Brown BL, Erickson LD (2015). Association between latent toxoplasmosis and cognition in adults: a cross-sectional study. Parasitology.

[82] Nance JP, Vannella KM, Worth D (2012). Chitinase dependent control of protozoan cyst burden in the brain. PLoS Pathog.

[83] Wang ZT, Harmon S, O’Malley KL, Sibley LD (2015). Reassessment of the role of aromatic amino acid hydroxylases and the effect of infection by Toxoplasma gondii on host dopamine. Infect Immun.

[84] Gulinello M, Acquarone M, Kim JH (2010). Acquired infection with Toxoplasma gondii in adult mice results in sensorimotor deficits but normal cognitive behavior despite widespread brain pathology. Microbes Infect.

[85] Haroon F, Händel U, Angenstein F (2012). Toxoplasma gondii actively inhibits neuronal function in chronically infected mice. PLoS One.

[86] Zhang W, Hao J, Liu R (2011). Soluble Aβ levels correlate with cognitive deficits in the 12-month-old APPswe/PS1dE9 mouse model of Alzheimer’s disease. Behav Brain Res.

[87] Lesné S, Kotilinek L, Ashe KH (2008). Plaque-bearing mice with reduced levels of oligomeric amyloid-beta assemblies have intact memory function. Neuroscience.

[88] Heppner FL, Ransohoff RM, Becher B (2015). Immune attack: the role of inflammation in Alzheimer disease. Nat Rev Neurosci.

[89] Sponaas AM, Freitas do Rosario AP, Voisine C (2009). Migrating monocytes recruited to the spleen play an important role in control of blood stage malaria. Blood.

[90] Sheel M, Engwerda CR (2012). The diverse roles of monocytes in inflammation caused by protozoan parasitic diseases. Trends Parasitol.

[91] London JA, Biegel D, Pachter JS (1996). Neurocytopathic effects of beta-amyloid-stimulated monocytes: a potential mechanism for central nervous system damage in Alzheimer disease. Proc Natl Acad Sci U S A.

[92] Akiyama H, Barger S, Barnum S (2000). Inflammation and Alzheimer’s disease. Neurobiol Aging.

[93] Lyman M, Lloyd DG, Ji X, et al. Neuroinflammation: The role and consequences. Neurosci Res 1–12. 2013. doi:10.1016/j.neures.2013.10.004 10.1016/j.neures.2013.10.004 24144733

[94] Ziv Y, Ron N, Butovsky O (2006). Immune cells contribute to the maintenance of neurogenesis and spatial learning abilities in adulthood. Nat Neurosci.

[95] Frenkel D, Huang Z, Maron R (2005). Neuroprotection by IL-10-producing MOG CD4+ T cells following ischemic stroke. J Neurol Sci.

[96] El Khoury J, Toft M, Hickman SE (2007). Ccr2 deficiency impairs microglial accumulation and accelerates progression of Alzheimer-like disease. Nat Med.

[97] Hawkes CA, McLaurin J (2009). Selective targeting of perivascular macrophages for clearance of beta-amyloid in cerebral amyloid angiopathy. Proc Natl Acad Sci U S A.

[98] Michaud J-P, Bellavance M-A, Préfontaine P, Rivest S (2013). Real-time in vivo imaging reveals the ability of monocytes to clear vascular amyloid beta. Cell Rep.

[99] Fiala M, Lin J, Ringman J (2005). Ineffective phagocytosis of amyloid-beta by macrophages of Alzheimer’s disease patients. J Alzheimers Dis.

[100] Prokop S, Miller KR, Drost N (2015). Impact of peripheral myeloid cells on amyloid-β pathology in Alzheimer’s disease-like mice. J Exp Med.

[101] Varvel NH, Grathwohl SA, Degenhardt K (2015). Replacement of brain-resident myeloid cells does not alter cerebral amyloid-β deposition in mouse models of Alzheimer’s disease. J Exp Med.

[102] Gensel JC, Nakamura S, Guan Z (2009). Macrophages promote axon regeneration with concurrent neurotoxicity. J Neurosci.

[103] Hohsfield LA, Humpel C (2015). Intravenous Infusion of Monocytes Isolated from 2-Week-Old Mice Enhances Clearance of Beta-Amyloid Plaques in an Alzheimer Mouse Model. PLoS One.

[104] Baruch K, Deczkowska A, Rosenzweig N, et al. PD-1 immune checkpoint blockade reduces pathology and improves memory in mouse models of Alzheimer’s disease. Nat Med. 2016. 1–5. doi:10.1038/nm.4022 10.1038/nm.4022 26779813

[105] Savage JC, Jay T, Goduni E (2015). Nuclear receptors license phagocytosis by trem2+ myeloid cells in mouse models of Alzheimer’s disease. J Neurosci.

[106] Collins JM, King AE, Woodhouse A (2015). The effect of focal brain injury on beta-amyloid plaque deposition, inflammation and synapses in the APP/PS1 mouse model of Alzheimer’s disease. Exp Neurol.

[107] Mildner A, Schlevogt B, Kierdorf K (2011). Distinct and Non-Redundant Roles of Microglia and Myeloid Subsets in Mouse Models of Alzheimer’s Disease. J Neurosci.

[108] Musiek ES, Holtzman DM (2015). Three dimensions of the amyloid hypothesis: time, space and “wingmen.”. Nat Neurosci.

[109] Jonsson T, Stefansson H, Steinberg S (2013). Variant of TREM2 associated with the risk of Alzheimer’s disease. N Engl J Med.

[110] Guerreiro R, Wojtas A, Bras J (2013). TREM2 variants in Alzheimer’s disease. N Engl J Med.

[111] Jones BM, Bhattacharjee S, Dua P (2014). Regulating amyloidogenesis through the natural triggering receptor expressed in myeloid/microglial cells 2 (TREM2). Front Cell Neurosci.

[112] Jiang T, Yu J-T, Zhu X-C, Tan L (2013). TREM2 in Alzheimer’s disease. Mol Neurobiol.

[113] Cantoni C, Bollman B, Licastro D (2015). TREM2 regulates microglial cell activation in response to demyelination in vivo. Acta Neuropathol.

[114] Wang Y, Cella M, Mallinson K, et al. TREM2 Lipid Sensing Sustains the Microglial Response in an Alzheimer’s Disease Model. Cell. 2015. 1–11. doi:10.1016/j.cell.2015.01.049 10.1016/j.cell.2015.01.049 PMC4477963 25728668

[115] Yamanaka M, Ishikawa T, Griep A (2012). PPARγ/RXRα-induced and CD36-mediated microglial amyloid-β phagocytosis results in cognitive improvement in amyloid precursor protein/presenilin 1 mice. J Neurosci.

[116] Koenigsknecht J, Landreth G (2004). Microglial phagocytosis of fibrillar beta-amyloid through a beta1 integrin-dependent mechanism. J Neurosci.

[117] Kagan JC, Horng T (2013). NLRP3 inflammasome activation: CD36 serves double duty. Nat Immunol.

[118] Frenkel D, Wilkinson K, Zhao L (2013). Scara1 deficiency impairs clearance of soluble amyloid-β by mononuclear phagocytes and accelerates Alzheimer’s-like disease progression. Nat Commun.

[119] Moore KJ, El Khoury J, Medeiros LA (2002). A CD36-initiated signaling cascade mediates inflammatory effects of beta-amyloid. J Biol Chem.

[120] Wilkinson K, El Khoury J (2012). Microglial scavenger receptors and their roles in the pathogenesis of Alzheimer’s disease. Int J Alzheimers Dis.

[121] Saido T, Leissring MA (2012). Proteolytic degradation of amyloid β-protein. Cold Spring Harb Perspect Med.

[122] Leissring MA, Farris W, Chang AY (2003). Enhanced proteolysis of beta-amyloid in APP transgenic mice prevents plaque formation, secondary pathology, and premature death. Neuron.

[123] Hoshino T, Murao N, Namba T (2011). Suppression of Alzheimer’s disease-related phenotypes by expression of heat shock protein 70 in mice. J Neurosci.

[124] Brkic M, Balusu S, Van Wonterghem E (2015). Amyloid β Oligomers Disrupt Blood-CSF Barrier Integrity by Activating Matrix Metalloproteinases. J Neurosci.

[125] Mukherjee A, Song E, Kihiko-Ehmann M (2000). Insulysin hydrolyzes amyloid beta peptides to products that are neither neurotoxic nor deposit on amyloid plaques. J Neurosci.

[126] Zhu S, Lai D-H, Li S-Q, Lun Z-R (2006). Stimulative effects of insulin on Toxoplasma gondii replication in 3T3-L1 cells. Cell Biol Int.

[127] Dantuma NP, Bott LC (2014). The ubiquitin-proteasome system in neurodegenerative diseases: precipitating factor, yet part of the solution. Front Mol Neurosci.

[128] Seifert U, Bialy LP, Ebstein F (2010). Immunoproteasomes preserve protein homeostasis upon interferon-induced oxidative stress. Cell.

[129] Orre M, Kamphuis W, Dooves S (2013). Reactive glia show increased immunoproteasome activity in Alzheimer’s disease. Brain.

[130] Kremer M, Henn A, Kolb C (2010). Reduced immunoproteasome formation and accumulation of immunoproteasomal precursors in the brains of lymphocytic choriomeningitis virus-infected mice. J Immunol.

[131] Mundt S, Engelhardt B, Kirk CJ, et al. Inhibition and deficiency of the immunoproteasome subunit LMP7 attenuates LCMV-induced meningitis. Eur J Immunol. 2015. n/a–n/a. doi:10.1002/eji.201545578 10.1002/eji.201545578 26464284

[132] Orre M, Kamphuis W, Osborn LM (2014). Isolation of glia from Alzheimer’s mice reveals inflammation and dysfunction. Neurobiol Aging.

[133] Neal LM, Knoll LJ (2014). Toxoplasma gondii profilin promotes recruitment of Ly6Chi CCR2+ inflammatory monocytes that can confer resistance to bacterial infection. PLoS Pathog.

