# Supplementary figures and images for: Chronic Toxoplasma gondii infection enhances β-amyloid phagocytosis and clearance by recruited monocytes
Source: Acta Neuropathol Commun. 2016 Mar 16;4:25. doi: 10.1186/s40478-016-0293-8 (PMC4793516; doi:10.1186/s40478-016-0293-8)

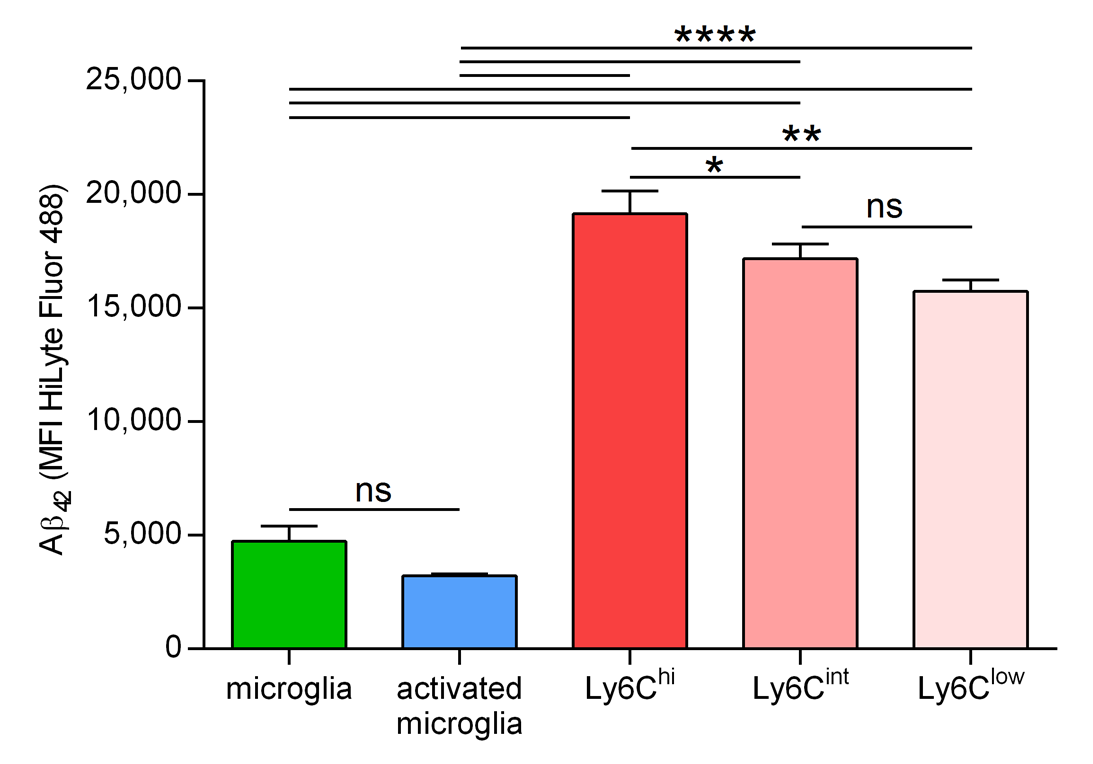

Supplement: Additional file 1: Figure S1. — Ex vivo Aβ42 uptake by cells from wildtype C57BL/6 mice. Ex vivo phagocytosis assay was performed with mononuclear cells isolated from C57BL/6 mouse brains. Populations were gated as described in Fig. 3a and Aβ42 uptake was quantified using imaging flow cytometry. Bars indicate the median fluorescence intensity (MFI) of each population and data are displayed as mean + SEM (n = 5). Significance levels (p values) determined by Fisher’s LSD test are indicated. ns, not significant, *p ≤ 0.05, **p ≤ 0.01, ****p ≤ 0.0001. (TIF 144 kb) [file 40478_2016_293_MOESM1_ESM.tif]
